# Natural killer cell phenotype is altered in HIV-exposed seronegative women

**DOI:** 10.1371/journal.pone.0238347

**Published:** 2020-09-01

**Authors:** Nancy Q. Zhao, Elena Vendrame, Anne-Maud Ferreira, Christof Seiler, Thanmayi Ranganath, Michel Alary, Annie-Claude Labbé, Fernand Guédou, Johanne Poudrier, Susan Holmes, Michel Roger, Catherine A. Blish

**Affiliations:** 1 Department of Medicine, Division of Infection Diseases and Geographic Medicine, Stanford University, Stanford, CA, United States of America; 2 Immunology Program, Stanford University, Stanford, CA, United States of America; 3 Department of Statistics, Stanford University, Stanford, CA, United States of America; 4 Centre de Recherche du CHU de Québec–Université Laval, Québec, Canada, Département de Médecine Sociale et Préventive, Université Laval, Québec, Canada, Institut National de Santé Publique du Québec, Québec, Canada; 5 Département de Microbiologie, Infectiologie et Immunologie de l‘Université de Montréal, Montréal, Canada, Service de maladies infectieuses et microbiologie, Hôpital Maisonneuve-Rosemont, Montréal, Canada; 6 Dispensaire IST, Cotonou, Bénin; 7 Laboratoire d’Immunogénétique, Centre de Recherche du Centre Hospitalier de l’Université de Montréal (CRCHUM), Montréal, Canada, Département de Microbiologie, Infectiologie et Immunologie de l‘Université de Montréal, Montréal, Canada; 8 Chan Zuckerberg Biohub, San Francisco, CA, United States of America; Emory University School of Medicine, UNITED STATES

## Abstract

Highly exposed seronegative (HESN) individuals present a unique setting to study mechanisms of protection against HIV acquisition. As natural killer (NK) cell activation and function have been implicated as a correlate of protection in HESN individuals, we sought to better understand the features of NK cells that may confer protection. We used mass cytometry to phenotypically profile NK cells from a cohort of Beninese sex workers and healthy controls. We found that NK cells from HESN women had increased expression of NKG2A, NKp30 and LILRB1, as well as the Fc receptor CD16, and decreased expression of DNAM-1, CD94, Siglec-7, and NKp44. Using functional assessments of NK cells from healthy donors against autologous HIV-infected CD4^+^ T cells, we observed that NKp30^+^ and Siglec-7^+^ cells had improved functional activity. Further, we found that NK cells from HESN women trended towards increased antibody-dependent cellular cytotoxicity (ADCC) activity; this activity correlated with increased CD16 expression. Overall, we identify features of NK cells in HESN women that may contribute to protection from HIV infection. Follow up studies with larger cohorts are warranted to confirm these findings.

## Introduction

Human immunodeficiency virus (HIV) remains a significant health problem, with 37.9 million people still living with HIV at the end of 2018 and an estimated 1.7 million new infections every year (www.who.int). Many advances have been made in the treatment and prevention of HIV. The advent of antiretroviral therapy (ART) has transformed HIV from a universally fatal disease into a manageable disease with near-normal life expectancy, and pre-exposure prophylaxis (PrEP) with antiretrovirals is highly effective in preventing HIV acquisition. However, ART use for treatment and prevention has serious limitations, including cost, side-effects and accessibility, making novel HIV prevention and treatment strategies desperately needed to halt the epidemic.

Highly HIV-exposed seronegative (HESN) individuals are a unique population who show a natural resistance to HIV acquisition despite repeated exposures. The study of these individuals has identified multiple correlates of protection from HIV acquisition, and a better understanding of these correlates could facilitate the design of innovative preventive measures and vaccine approaches.

Natural killer (NK) cells are able to quickly and rapidly respond to viral infections and their function is determined by the combinatorial signaling of inhibitory and activating receptors expressed on the cell surface [1]. NK cells have been implicated in early immune responses to HIV infection (reviewed in [2]). Although traditionally this was thought to be a non-antigen-specific response, recent data have shown that NK cells may also be capable of generating memory-like responses to viral antigens, including HIV [3–7]. NK cells expand during the early stages of HIV infection [8,9], and respond to HIV *in vivo* and *in vitro* [10–13]. NK cell-mediated antibody-dependent cytotoxicity (ADCC) has also been linked to slower disease progression [14,15] and, when combined with specific human leukocyte antigen (HLA) alleles, certain killer immunoglobulin-like receptors (KIR) were associated with slower disease progression [16–18], and elite control of HIV [19].

Increasing evidence suggests that specific NK cell features can also confer protection from HIV acquisition. Genetic studies revealed that the presence of specific KIR/HLA combinations may contribute to protection of HESN from HIV infection via intravenous or sexual routes [20–26]. Additionally, a less diverse and more flexible NK cell receptor repertoire has been associated with lower risk of acquiring HIV in sexually exposed Kenyan women [27]. NK cell function has also been linked with HIV protection. Increased NK cell activation has been seen in HIV-exposed intravenous drug users [28–30] and in sexually exposed HESN [31,32]. Additionally, NK cell-mediated ADCC has been linked to vaccine-induced protection from HIV infection [33].

Prior studies of immune correlates of HIV protection in Beninese HESN women revealed a low inflammatory immune profile in the blood and genital tract. In fact, low levels of soluble B lymphocyte stimulator (BLyS)/BAFF were detected in the blood and cervicovaginal lavages (CVL) [34,35] and low levels of pro-inflammatory cytokines in the CVL of these HESN women [36,37]. This raised the possibility that HIV resistance in these women may be the result of a balance between strong innate immune responses and low inflammatory conditions and fewer HIV target cells at the exposure site [38]. To study how innate NK cell responses may contribute to protection in these women, we used cytometry by time of flight (CyTOF) to profile the NK cell receptor repertoire of 20 HESN women and 10 healthy controls from this cohort, and performed NK cell functional assessments.

## Materials and methods

### Study participants

Cryopreserved peripheral blood mononuclear cells (PBMCs) were obtained from Beninese women, as previously described [35,39]. HESN were enrolled from a female sex-worker clinic; HESNs were women who remained HIV-uninfected after at least 3.5 years of sex work. Healthy unexposed HIV-1-seronegative women were enrolled from a general health clinic and were either married or living with a male partner. PBMCs were obtained from 20 HESN and 10 healthy women. Written informed consent was obtained from all women. The study was approved by the Comité National Provisoire d’Éthique de la Recherche en Santé in Cotonou and the Centre Hospitalier de l’Université de Montréal (CHUM) Research Ethics Committees. Demographics from the study participants are summarized in Table 1.

**Table 1:** Study group demographics.

	Healthy Women n = 10	HESN n = 20
**Age, Mean (SD), Years**	34 (7)	35 (9)
**Years of sex work at study visit, Mean (SD)**	n/a	5.1 (0.9)
**Use of oral contraceptives, Number of subjects**	0	1
**Vaginal douching, Number of Subjects**	n/a	20
**Number of clients in last 7 days, Mean (SD)**	n/a	15 (18)
**Condom always used with client in the last 7 days, Number of subjects**	n/a	16
Sexually Transmitted Infections*, Number of Subjects (%)	1 (10))	1 (5)
Vaginal Candidiasis, Number of Subjects (%)	1 (10)	3 (15)
Bacterial Vaginosis, Number of Subjects (%)	9 (90)	17 (85)

n = number of subjects, SD = standard deviation. There were no significant differences in mean age between groups, at a p-value threshold of 0.05 using the Mann-Whitney-Wilcoxon test.

*There were no significant differences in frequency of sexually transmitted infections, vaginal candidiasis or bacterial vaginosis between groups, at a p-value threshold of 0.05 using the Fisher Exact Test. Sexually transmitted infections included: one woman with Chlamydia in the HESN group and one woman with both Chlamydia and Gonorrhea in the healthy women group. There were no cases of Syphilis or Trichomoniasis.

For functional validation of NK markers (Fig 3), leukoreduction system chambers from 20 anonymous, healthy donors were obtained from the Stanford Blood Bank. PBMCs were isolated by density gradient centrifugation using Ficoll-Paque PLUS (GE Healthcare, Chicago, IL, USA), and cryopreserved in 10% DMSO (Sigma Aldrich, St Louis, MO, USA) and 90% heat-inactivated fetal bovine serum (FBS) (Thermo Fisher Scientific, Waltham, MA, USA).

### Mass cytometry for NK cell profiling of HESN and healthy Beninese women

NK cells were purified from PBMCs by magnetic-activated isolation via negative selection (Miltenyi, Bergisch Gladbach, Germany) and stained for mass cytometry as described previously [40], using CyTOF Panel 1 (S1 Table). All antibodies were conjugated using MaxPar® ×8 labeling kits (Fluidigm, South San Francisco, CA, USA). To ensure antibody stability over time, antibody panels were lyophilized into single-use pellets prior to use (Biolyph, Chaska, MN, USA). Briefly, NK cells were plated in 96-deep-well plates, resuspended in 25 mM cisplatin (Enzo Life Sciences, Farmingdale, NY, USA) for 1 minute and then quenched with 100% FBS. Cells were washed twice and stained for 30 minutes at 4°C, fixed (BD FACS Lyse, BD Biosciences, Franklin Lakes, NJ, USA), permeabilized (BD FACS Perm II), and stained with intracellular antibodies for 45 minutes at 4°C. Cells were resuspended overnight in iridium intercalator (Fluidigm) in 2% paraformaldehyde in PBS. Cells were washed 1 time in PBS and 2 times in water and resuspended in 1× EQ Beads (Fluidigm) before acquisition on a Helios mass cytometer (Fluidigm).

### Mass cytometry data pre-processing

The open source statistical package R (https://www.r-project.org/) was used for all statistical analyses [41]. Mass cytometry data were bead-normalized with EQ Beads (Fluidigm) prior to subsequent analyses, using the *Premessa* package (https://github.com/ParkerICI/premessa) [42]. Data were first visualized with FlowJo v10.5.3 (Tree Star, Woodburn, OR, USA). Markers noted to have poor staining were excluded from subsequent analyses (FAS-L, Ki-67, KIR2DS2 and CXCR6 for Panel 1; CD11a and KIR2DS2 for Panel 2). Serial negative gating was used to identify NK cells, as described [40] (S1 Fig). Normalized signal intensities were transformed using the inverse hyperbolic sine (asinh) function with a cofactor equal to 5 to account for heteroskedasticity, prior to subsequent analyses. The data supporting this publication is available at ImmPort (https://www.immport.org) under study accession SDY1647.

### Multidimensional scaling and generalized linear model

**Fig 1:**
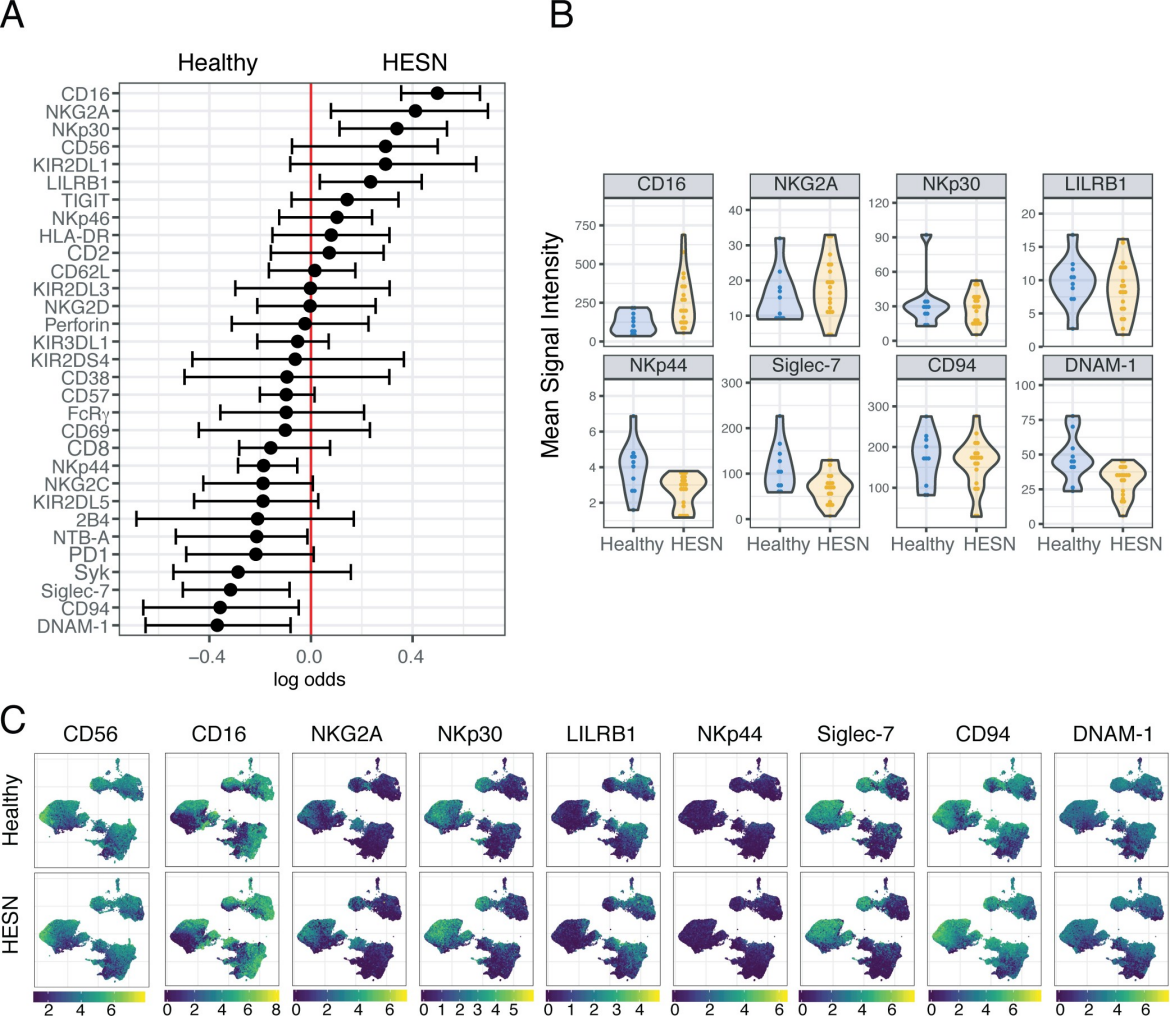
NK cells from HESN women are phenotypically distinct from healthy women. **(A)** A generalized linear model with bootstrap resampling was used to identify receptors predictive of NK cells from HESN (n = 16) or healthy (n = 9) women. For each marker, the 95% confidence interval is represented by the line surrounding the point estimate. **(B)** Mean signal intensity (MSI) of CD16, NKG2A, NKp30 and LILRB1 (significant predictors of the HESN group identified in **A**; top), and NKp44, Siglec-7, CD95 and DNAM-1(significant predictors of the healthy group; bottom). **(C)** UMAP visualization of all NK cells from HESN and healthy donors, colored by expression of the same markers as in **B** as well as CD56. Scales show asinh-transformed channel values.

The custom-made package *CytoGLMM* was used to create an exploratory multidimensional scaling (MDS) projection (Fig 1A) and to build a confirmatory generalized linear model (GLM). A GLM with bootstrap resampling (n = 100 bootstraps) was used to identify markers predictive of HESN or healthy (Fig 1B) [43]. Samples with cell numbers smaller than 1000 were excluded from analyses; the number of subjects used for each analysis is specified in the figure legend. Mean signal intensities of the markers identified by the GLM were then compared between HESN and healthy women using the Wilcoxon rank-sum test.

### Clustering

Unsupervised clustering was performed using the R package *CATALYST* [44,45]. The clustering method in this package uses the *FlowSOM* algorithm [46] to first generate 100 high-resolution clusters, followed by a metaclustering step with the *ConsensusClusterPlus* algorithm [47] to regroup these high-resolution clusters into metaclusters. Default parameters were used for clustering, and the number of metaclusters (8) was selected based on the delta area plot provided. To test for differential abundance of clusters between healthy and HESN, the *diffcyt-DA-GLMM* method from the *diffcyt* package was used which computes tests using a Generalized Linear Mixed Model (GLMM).

### NK cell functional experiments

NK cells and CD4^+^ T cells from healthy blood bank donors were separately purified from PBMCs by negative selection (Miltenyi). NK cells were cultured with 300 IU/ml recombinant human IL-2 (R&D Systems, Minneapolis, MN, USA) for 72h, and CD4^+^ T cells were activated and infected with Q23-FL, a HIV-1 clone from early, subtype A infection [48], as previously described [49]. 5x10^5^ NK cells were co-cultured with 2x10^6^ HIV-infected CD4^+^ T cells (1:4 effector:target ratio) for 4h in the presence of brefeldin A (eBioscience, San Diego, CA, USA), monensin (eBioscience), and anti-CD107a-APC (Biolegend, San Diego, CA, USA). At the end of co-culture, cells were stained for mass cytometry as described above, using the CyTOF Panel 2 (S2 Table). To maintain antibody stability and consistency in staining, antibodies in CyTOF panel 2 were pre-mixed into separate surface and ICS cocktails, as indicated in S2 Table, aliquoted and frozen at -80°C until use.

### ADCC assay

NK cells were purified from PBMCs from healthy and HESN samples by negative selection using the NK Cell Isolation Kit (Miltenyi, Bergisch Gladbach, Germany). 1x10^5^ NK cells were mixed with 4x10^5^ CD20^+^ Raji cells (ATCC CCL-86, used at passage 9–11), in the presence or absence of 1μg/ml Rituximab (non-fucosylated human CD20 IgG1 antibody, Invivogen, San Diego, CA, USA). Co-cultures were incubated for 4 hours at 37°C, in RP10 with brefeldin A (eBioscience), monensin (eBioscience), and anti-CD107a-APC-H7 (BD Biosciences, clone H4A3). At the end of co-culture, cells were stained with Zombie Aqua Fixable Viability dye (Biolegend), and surface stained with anti-CD3-PE (Biolegend, clone UCHT1), anti-CD16-FITC (Biolegend, clone 3G8), anti-CD56-PE-Cy7 (Biolegend, clone HCD56) and anti-CD19-APC (Biolegend, clone HIB19). Cells were subsequently fixed with FACS Lyse (BD Biosciences), permeabilized with FACS Permeabilization Buffer 2 (BD Biosciences), and stained for intracellular cytokines with anti-IFN-ɣ-V450 (BD Biosciences, clone B27) and TNF-ɑ-BV650 (Biolegend, clone MAb11). Cells were analyzed by flow cytometry using an Aurora spectral cytometer (Cytek Biosciences, Fremont, CA, USA), and data analysis was performed using FlowJo version 10.1 (Tree Star). Background subtracted % positive for each functional marker = % positive for functional marker in NK + Raji + Rituximab well—% positive for functional marker in NK + Raji—Rituximab well.

### CD16 genotyping of HESN and healthy Beninese women

DNA from 105 PBMCs from healthy and HESN samples was extracted using the DNeasy Blood and Tissue Kit (Qiagen, Hilden, Germany). The region of the FCGR3A gene containing the 158V/F polymorphism was amplified using nested PCR, with primers and cycling conditions as previously described [50], using Q5 High Fidelity DNA Polymerase (New England Biolabs, Ipswitch, MA, USA). PCR cleanup and Sanger sequencing were performed by Elim Biopharm (Hayward, CA, USA). Each sample underwent PCR and sequencing in duplicate. All samples, together with a reference FCGR3A sequence (NCBI Reference Sequence: NG_009066.1), were aligned in Geneious Prime Version 2020.0.4 (Biomatters, Auckland, New Zealand). The polymorphism variants were identified using analysis of the chromatograms at nucleotide position 5093—T/T corresponded to the F/F phenotype, T/G to the V/F phenotype, and G/G to the V/V phenotype.

## Results

### Peripheral blood NK cells from HESN are phenotypically distinct from those of healthy women

To determine the effect of HIV-1 exposure on the NK cell phenotype, we used a generalized linear model (GLM) with bootstrap resampling to compare NK cells from HESN and healthy women and identify predictors of either group. Using this strategy, we found that increased expression of CD16, NKG2A, NKp30 and LILRB1 were the strongest predictors of HESN, and that DNAM-1, CD94, Siglec-7, and NKp44 were the strongest predictors of the healthy women group (Fig 1A). To confirm these results, we compared the mean signal intensity (MSI) for the top 4 NK cell markers predictive of each of the study groups and we observed that HESN had an increased MSI for CD16 and a decreased MSI for DNAM-1, Siglec-7 and NKp44 (Fig 1B). To better understand the subsets of cells on which these markers were expressed, we visualized the expression profiles of these markers by simplifying the multidimensional space using UMAP (Fig 1C). By overlaying the expression of CD56 and CD16, we identified the canonical CD56^−^, CD56^dim^, and CD56^bright^ NK cell subsets. For our markers of interest identified by the GLM, we saw that the expression of these markers was globally distributed, and that markers predictive of each arm of our study were not co-expressed on a specific NK cell subset. To further explore the effect of HIV exposure on NK cell phenotype, we compared the frequency of the canonical CD56^−^, CD56^dim^, and CD56^bright^ NK cell subsets between our groups and we found no significant differences (S2 Fig).

### Peripheral blood NK cells from HESNs do not form distinct subsets

**Fig 2:**
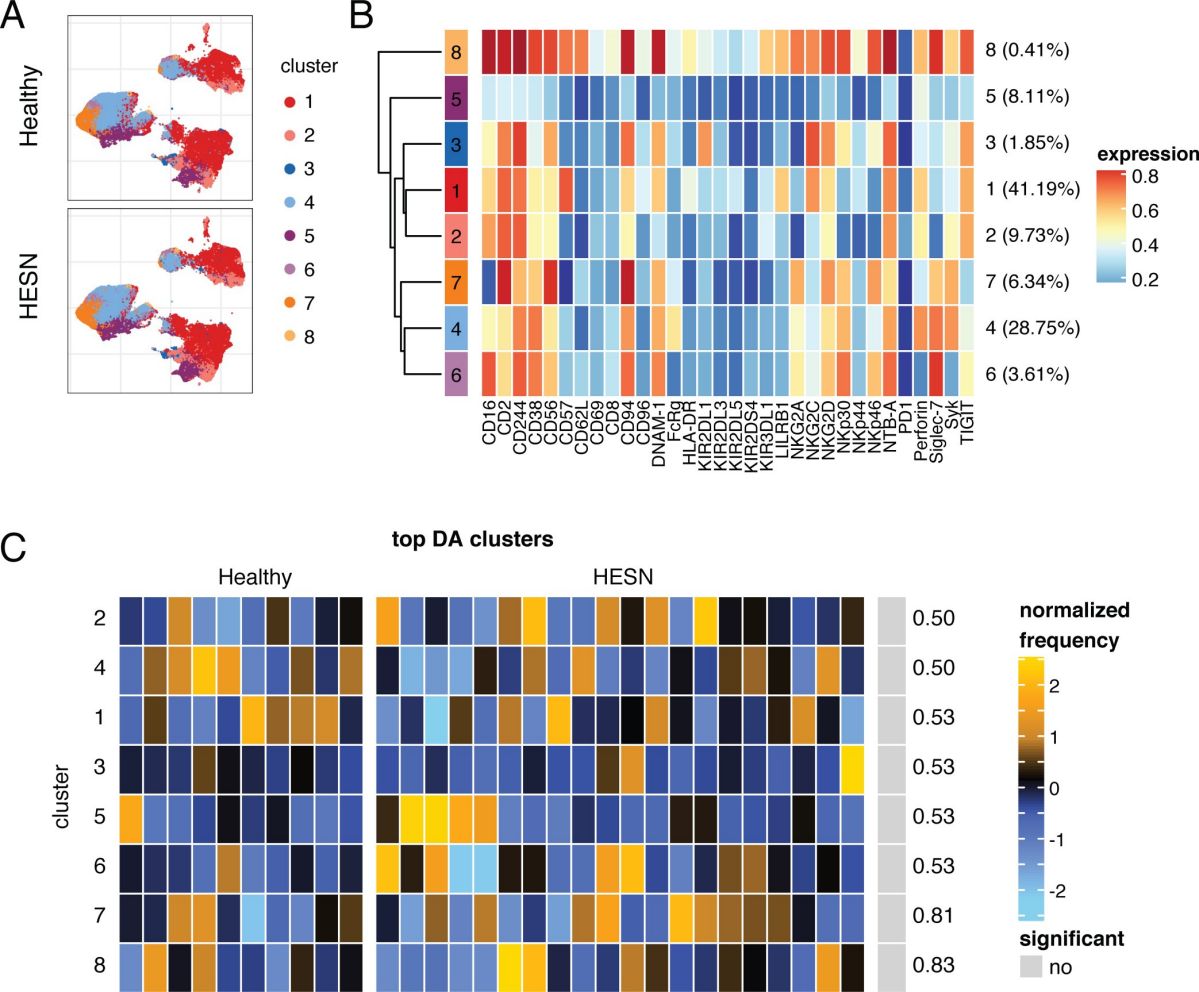
NK cells from HESNs do not belong to distinct clusters. **(A)** UMAP visualization of all NK cells from HESN (n = 20) and healthy women (n = 10), colored by metacluster identity generated by CATALYST clustering method. **(B)** Heatmap of scaled mean expression of all NK markers profiled, for each cluster 1 to 8. The overall abundance of each cluster, as a percent of total cells, is displayed to the right of the heatmap. **(C)** Heatmap of the relative abundance of each cluster between the healthy (left) and HESN (right) groups. Each column represents a single donor and shows the normalized frequency of cells belonging to each cluster. The normalized frequencies are proportions that were first scaled with arcsine-square-root transformation and then z-score normalized in each cluster (light blue showing under-representation and light yellow showing over-representation). Adjusted p-values for differential abundance (DA) tests by GLMM, between the two groups, are displayed on the right.

To confirm that the differences in NK receptor expression observed in HESN compared to healthy women did not occur on a unique subset of NK cells, we used the CATALYST package to cluster all NK cells from both the healthy and HESN groups. This method identified 8 clusters of NK cells (Fig 2A and 2B). We then tested for differential abundance of each of these NK cell clusters between the two groups, using a generalized linear mixed model (GLMM). No differentially abundant clusters were found (Fig 2C), indicating that the changes in NK cell receptor expression in HESNs reflect global changes in expression patterns of certain receptors rather than the development of a unique subset of NK cells.

### CD16^-^, NKp30^+^ and Siglec-7^+^ NK cells from healthy donors show increased HIV-specific NK cell responses

**Fig 3:**
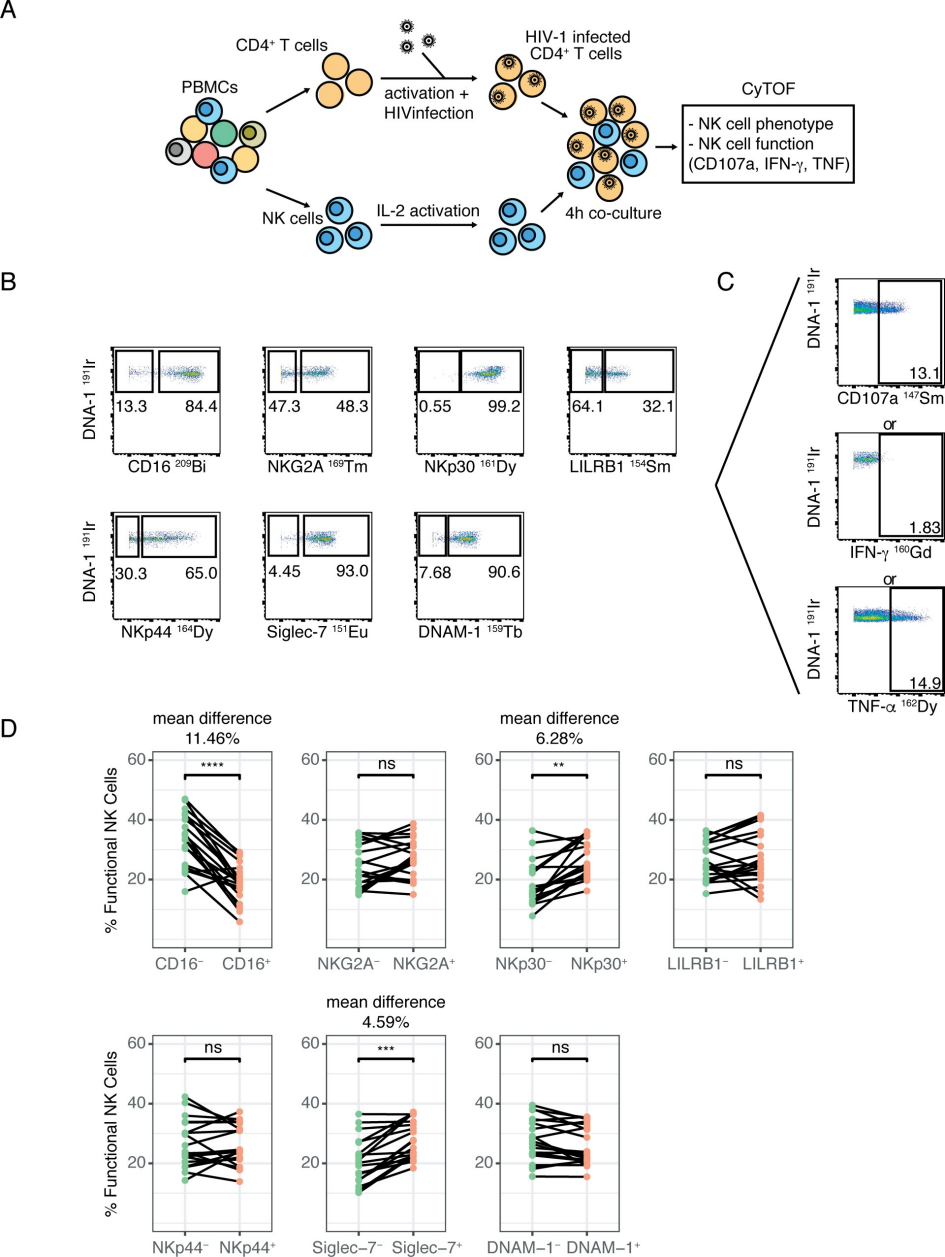
HIV-specific NK cell functional activity is increased in NKp30^+^ and Siglec-7^+^ NK cells. **(A)** Experiment schematic: NK cells and CD4^+^ T cells were isolated from healthy blood bank donors (n = 20). CD4^+^ T cells were infected with Q23. NK cells and CD4^+^ T cells were co-cultured for 4 hours at a 1:4 effector:target ratio, and phenotype and NK cell function measured by CyTOF. **(B)** Example of CyTOF stain and gating for NKG2A, NKp30, NKp44, DNAM-1, Siglec-7 and NKp44. **(C)** Example of functional activity of NK cells, measured by CD107a, IFN-ɣ and TNF-ɑ production. **(D)** Summary data comparing functional activity (measured as frequency of CD107a+ or IFN-ɣ^+^ or TNF-ɑ^+^ NK cells) of NKG2A^+^ and NKG2A^-^, NKp30^+^ and NKp30^-^, LILRB-1^+^ and LILRB1^-^, DNAM-1^+^ and DNAM-1^-^, SIglec-7^+^ and Siglec7^-^, and NKp44^+^ and NKp44^-^ NK cells (n = 20). For markers whose positive and negative populations have a statistically significant difference in functional activity, the mean difference is shown above the plot. ** = p ≤ 0.01, *** = p ≤ 0.001, **** = p ≤ 0.0001, ns = not significant, by paired Wilcoxon signed-rank test, adjusted using the Benjamini-Hochberg method.

The GLM identified CD16, NKG2A, NKp30 and LILRB1 as predictors of the HESN group and DNAM-1, Siglec-7, CD94 and NKp44 as predictors of the healthy group (Fig 1B). To better characterize the role of these markers, we used CyTOF to investigate the ability of NK cell subpopulations expressing these markers to respond to HIV-infected CD4^+^ T cells *in vitro* (Fig 3A). We purified NK cells and autologous CD4+ T cells from PBMCs of healthy, HIV-uninfected blood bank donors. We then infected the CD4+ T cells with HIV and co-cultured NK cells with HIV-infected CD4+ T cells. After gating on positive and negative expression for each marker of interest (Fig 3B), we assessed overall functional activity, measured as the fraction of CD107a^+^ or IFN-ɣ^+^ or TNF-α^+^ NK cells, for each positive and negative population (Fig 3C). CD94 was excluded from this analysis—CD94 forms a heterodimer with both the inhibitory receptor NKG2A and the activating receptor NKG2C but has no direct signaling activity [51]; and was hence not included in CyTOF Panel 2. With this approach, we found that CD16^+^ NK cells showed significantly lower function compared to CD16^-^ NK cells. Additionally, NKp30^+^ NK cells showed increased response compared to NKp30^-^ NK cells. Similarly, we found that Siglec-7^+^ NK cells showed increased response compared to Siglec-7^-^ NK cells (Fig 3D). When we assessed functional activity by each single functional marker (CD107a, IFN-ɣ or TNF-α), we found that expression of each individual functional marker demonstrated the same trend as overall functional activity for CD16, NKp30 and Siglec-7 (S3 Fig). However, DNAM-1^+^ NK cells also showed increased cytokine production (IFN-ɣ or TNF-α) but decreased degranulation (CD107a) compared to DNAM-1^-^ NK cells, but no difference in overall function (Fig 3D, S3 Fig). We did not find significant differences in HIV responses between cells expressing NKG2A, LILRB1, or NKp44.

### Peripheral blood NK cells from HESN trend towards increased ADCC cytotoxic activity

As CD16 expression was the strongest predictor identified in our GLM analysis, with higher expression in HESN women compared to healthy women (Fig 1A), we sought to better understand the potential role of CD16 in these differences. Although CD16^-^ NK cells from healthy donors had increased functional activity compared to CD16^+^ in response to HIV-infected cells (Fig 3D), this may be due to downregulation of CD16 that is known to occur in activated NK cells, particularly those expressing CD107a and IFN-ɣ, even in the presence of stimulation that does not directly involve CD16 [52]. As such, we wanted to more directly address its role in NK cell responses. As CD16 is a Fcɣ receptor (FcɣRIIIa), we focused on the impact of differential CD16 expression on ADCC activity in NK cells.

**Fig 4:**
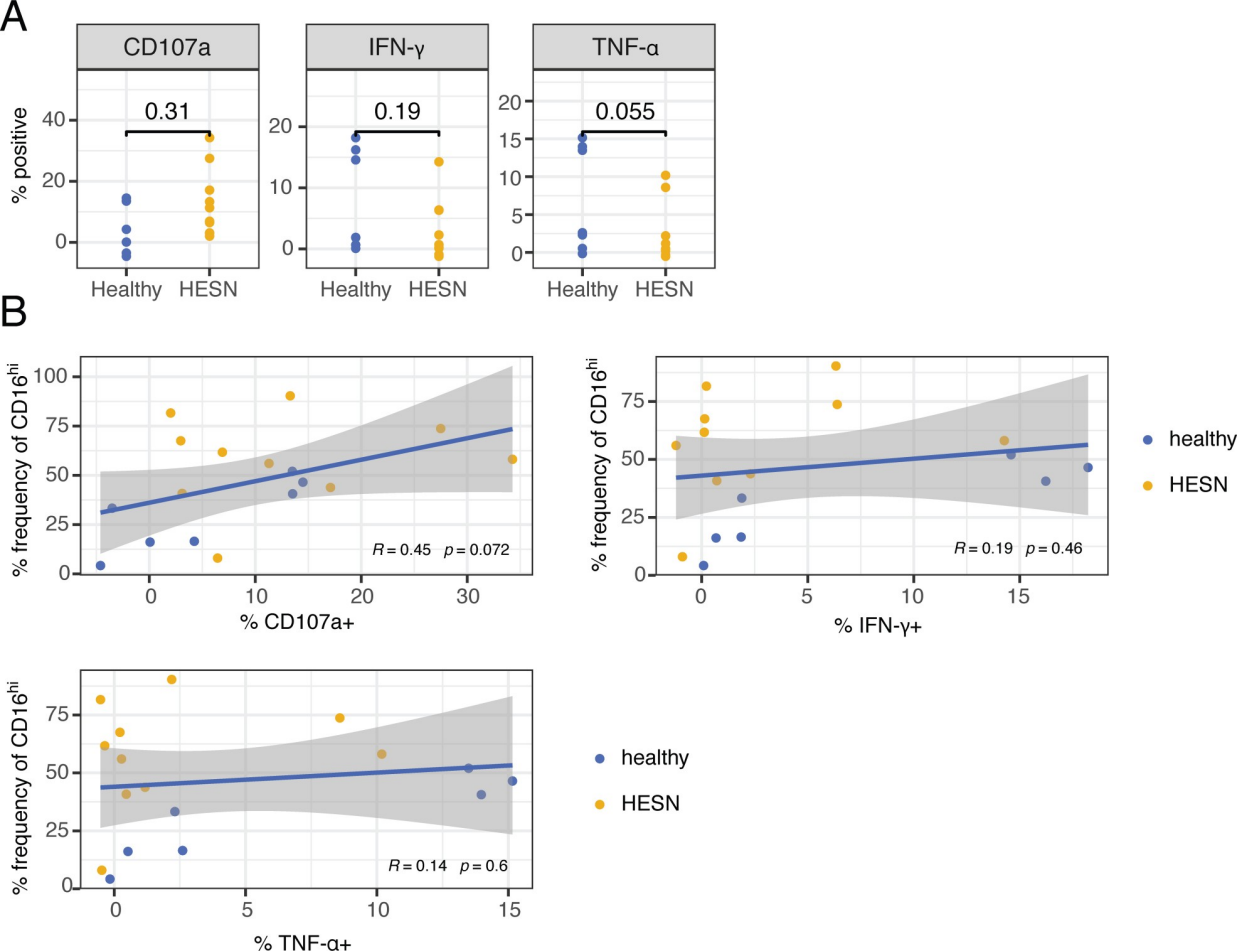
Differential CD16 expression on NK cells between healthy and HESNs impacts ADCC activity. **(A)** Background-subtracted frequency of cells positive for CD107a, IFN-γ, and TNF-ɑ in NK cells from healthy (n = 7) and HESN (n = 10) donors in an *in vitro* Rituximab-mediated ADCC assay with CD20^+^ Raji target cells. Exact p-values by unpaired Wilcoxon signed-rank test are shown for each plot. **(B)** Pearson correlation between percentage of CD16^hi^ cells (in the CyTOF profiling of NK cells) and each of the functional markers CD107a, IFN-γ, and TNF-ɑ (in the *in vitro* ADCC assay) for each donor in both healthy (n = 7) and HESN (n = 10) groups combined. Pearson correlation coefficient (R) and exact p-value is shown for each plot.

To determine if there are differences in intrinsic ADCC ability of NK cells between healthy and HESN women, we used an *in vitro* Rituximab-mediated ADCC assay with CD20^+^ Raji cells as target cells for individuals in whom an additional aliquot of PBMCs was available for functional assessment. We observed robust ADCC activity in the presence of Rituximab and Raji target cells (S4 Fig). To account for differences in baseline NK cell activity between women, we subtracted the percent positive for each functional marker in the co-cultures without Rituximab. While we were limited in the number of subjects we were able to evaluate, we found a trend of increased cytotoxic CD107a^+^ NK cells in the HESNs compared to the healthy controls. In contrast, the expression of cytokines IFN-ɣ and TNF-ɑ trended towards a decrease in the HESN group (Fig 4A).

To further understand the impact of CD16 expression on ADCC responses, we correlated the percentage of CD16^hi^ cells from each woman, as identified by our initial CyTOF screen, with the percentage of expression of each functional marker in the *in vitro* ADCC assay. There was a trend towards a correlation between the percentage of CD16^hi^ cells with CD107a in the ADCC assay, but not with IFN-ɣ and TNF-ɑ (Fig 4B). Together, these data suggest that HESNs had higher expression of CD16 that may be associated with increased ADCC activity by cytotoxicity.

## Discussion

HESN individuals provide insight to protective mechanisms against HIV acquisition, and increased NK cell activity has been observed in multiple studies of HESN subjects [28–30,53]. To characterize the phenotypic features of these NK cells in HESN subjects and gain insight into potential mechanisms of protection, we used mass cytometry to profile NK cell phenotype in a cohort of Beninese HESN women and healthy HIV-uninfected controls. We observed differences in expression of activating and inhibitory NK cell receptors between HESN and healthy women, including receptors such as Siglec-7 and NKp30, and NK cells expressing these receptors may have differential HIV-targeting activity. In addition, we found that increased CD16 expression on NK cells was associated with a trend of increased ADCC activity by cytotoxicity in the HESN group compared to healthy.

Previous studies have yielded consistent evidence of heightened NK cell activity in HESN individuals, including increased activation and functional activity of NK cells at baseline [28–32,53]. Studies of intravenous drug users reported an increase in expression of the activation marker CD69 in *ex vivo* NK cells in HESN individuals [28,30]. Conversely, one study reported a decrease of CD69 expression in NK cells from HESN women after IL-2 stimulation [54]. We did not observe an increase or decrease in CD69 expression in *ex vivo* NK cells from HESN women (Fig 1B). This may be a result of the lack of use of IL-2 stimulation in our *ex vivo* study, or of differential immune protection pathways at mucosal surfaces in our cohort of commercial sex workers, compared to in blood for intravenous drug users. Instead, the strongest phenotypic shift we observed in HESN individuals was enhanced expression of CD16 on NK cells, which has not been previously reported, but could have important functional implications. While our sample size was a limiting factor, we observed a trend of heightened ability of NK cells from HESN individuals to mediate ADCC.

ADCC is a potent effector mechanism of NK cells, mediated by virus-specific antibodies binding to CD16 (FcγRIIIa). The engagement of CD16 is a trigger of NK cell activation and cytolysis, leading to killing of antibody-bound infected cells [55]. ADCC activity is known to provide a protective benefit in disease progression in HIV-infected individuals—ADCC antibody titers correlates positively with CD4 count and negatively with viral load [56,57], and elite controllers have higher ADCC activity compared to viremic individuals [58]. In the female genital tract, the presence of antibodies that are capable of mediating ADCC against HIV gp120 is associated with reduced cervical viral load [59], suggesting that ADCC may act as a mechanism of defense against HIV in this setting. Our finding that NK cells from HESN women have a trend of increased ADCC activity may be particularly relevant as HIV gp41-reactive IgG1 antibodies have been found in CVL samples from women in this cohort [34]. Although no difference in ADCC-mediating antibodies have been observed in this cohort between HESN and healthy women [60], differences in ADCC activity at the level of effector cells could contribute to protection. However, as our observed increases in ADCC activity were small and not statistically significant, further work with larger HESN cohorts would provide more insight on this potential mechanism of protection.

One of the known mechanisms by which CD16 expression, as well as ADCC activity, can differ is due to a polymorphism at residue 158 of the CD16 protein; possessing at least one valine (V) at this residue, instead of phenylalanine (F), leads to increased cell surface CD16 expression as well as augmented Rituximab-mediated ADCC activity [61]. We genotyped samples of HESN and healthy Beninese women for this polymorphism, and, while we were underpowered to detect population-level genetic differences using this set of samples, we did not observe a skewing in distribution of CD16 variants (V/V, V/F and F/F) in the healthy compared to HESN group (S5 Fig. In addition, we did not observe differences in expression of other NK cell receptors such as NKG2D (Fig 1A) that are known to contribute to ADCC activity [62].

Aside from CD16, we found that NK cells from HESN subjects also had increased expression of NKG2A, NKp30 and LILRB1. Many of these receptors have been implicated in NK cell targeting of HIV. NKG2A-expressing NK cells have increased functional activity against HIV-infected cells [63,64], although we did not consistently observe this effect in our *in vitro* co-cultures (Fig 3D). In addition, we found that NKp30^+^ NK cells also had increased expression of functional markers against autologous HIV-infected CD4 T cells, compared to NKp30^-^ (Fig 3D). Similarly, LILRB1^+^ (CD85j^+^) NK cells have been found to exert potent suppression of HIV-infected dendritic cells [65], although we did not observe an increased functional response of LILRB1^+^ NK cells to HIV-infected CD4 T cells (Fig 3D). Collectively, it is possible that the increased expression of these markers on NK cells from HESN women represent NK cells that have increased functional capacity to target HIV in different settings.

In studies of HESN subjects in comparison with healthy, unexposed controls, it remains difficult to distinguish whether observed changes in immune cell phenotype and function represent potential mechanisms of protection against HIV acquisition, or markers of exposure to HIV. Indeed, some of the phenotypic changes we observed on NK cells from HESN women may represent the latter—for example, Siglec-7 expression on NK cells is also strongly reduced in viremic HIV-1-infected individuals, but not long term non progressors, and this downregulation is associated with NK cell dysfunction [66]. In addition, Siglec-7^-^ cells have been reported to have reduced functional capacity compared to Siglec-7^+^ NK cells [67], consistent with our findings (Fig 3D). Our observation of decreased Siglec-7 expression on NK cells from HESN women thus is unlikely to represent a protective mechanism against HIV acquisition.

Another consideration is that epidemiological differences between HESN and healthy women, in addition to HIV exposure, may drive NK cell changes observed in this study. Overall, the measured epidemiological parameters were well balanced (Table 1), with no differences in the rate of sexually transmitted infection, vaginal candidiasis or bacterial vaginosis between groups. Nevertheless, as commercial sex workers, HESN women are more exposed to semen from multiple partners. Semen can induce a local mucosal inflammatory response [68], thus could also potentially mediate NK cell activation.

There are several limitations to our study. Due to the difficulty in obtaining well-curated HESN cohorts, we had limited sample availability for follow-u\p studies on ADCC activity (10 and 7 HESN and healthy women respectively), and were thus underpowered to detect potential differences. Further studies, with larger HESN cohorts, are warranted, as mentioned above. We were only able to profile women exposed to HIV via sexual exposure; HIV exposure via intravenous drug use and in men may lead to differing outcomes.

In conclusion, we have shown that HESN women have increased expression of CD16, and trend towards increased ADCC activity, compared to healthy controls. In addition, they possess NK cells with increased expression of NKp30, NKG2A and LILRB1, all NK cell receptors whose expression is associated with improved anti-HIV activity. These may present potential mechanisms of NK-mediated protection against HIV acquisition in HESN women, and warrant follow-up in additional, larger studies.

## Supporting information

S1 Fig Gating schemes. Intact, bead and event-length gates ensure successful gating to single cells. Cisplatin stain was performed as Live/Dead stain. (A) Serial negative gating to NK cells for CyTOF Panel 1. T cells and B cells were excluded using CD3, and CD19. Monocytes were excluded by negative gating on CD4 and CD14/CD33 and by further negative gating of CD56^-^/HLA-DR^bright^ cells. CD56 and CD16 were used to identify NK cells. (B) Serial negative gating to NK cells for CyTOF Panel 2. T cells in the co-culture were excluded using CD3. CD56 and CD16 were used to identify NK cells.
(TIF)

S2 Fig Frequency of CD56 subsets does not differ between HESN and healthy women. **(A)** An example of gating strategy for classical NK cell subpopulations (CD56^-^, CD56^dim^ and CD56^bright^) in one healthy and one HESN woman. **(B)** Frequency of CD56^-^, CD56^dim^ and CD56^bright^ NK cells in healthy and HESN women. ns = non-significant.
(TIF)

S3 Fig HIV-specific NK cell functional activity of different NK cell subpopulations. Summary data comparing the frequency of CD107a^+^, IFN-ɣ^+^ and TNF-ɑ^+^ NK cells of NKG2A^+^ and NKG2A^-^, NKp30^+^ and NKp30^-^, LILRB-1^+^ and LILRB1^-^, DNAM-1^+^ and DNAM-1^-^, SIglec-7^+^ and Siglec7^-^, and NKp44^+^ and NKp44^-^ NK cells (n = 20). * = p ≤ 0.05 ** = p ≤ 0.01, *** = p ≤ 0.001, **** = p ≤ 0.0001, ns = not significant, by paired Wilcoxon signed-rank test, adjusted using the Benjamini-Hochberg method.
(TIF)

S4 Fig Rituximab-mediated ADCC assay allows identification of robust ADCC responses. Frequency of cells positive for CD107a, IFN-γ, and TNF-ɑ in NK cells from healthy (n = 7) and HESN (n = 10) donors in an in vitro Rituximab-mediated ADCC assay with CD20+ Raji target cells, in the absence (left) or presence (right) of Rituximab (anti-CD20). Exact p-values by unpaired Wilcoxon signed-rank test are shown for each plot.
(TIF)

S5 Fig CD16 genotyping of healthy and HESN women. Frequency of each CD16 variant (F/F, V/F and V/V) in the healthy (n = 7) and HESN (n = 10) groups. Genotyping was performed by Sanger sequencing of the CD16 gene in the region containing the polymorphism. No significant difference in the frequencies between the two groups was found by Fisher’s exact test.
(TIF)

S1 Table CyTOF Panel 1. (DOCX)

S2 Table CyTOF Panel 2. (DOCX)
