# Supplementary figures and images for: Natural killer cell phenotype is altered in HIV-exposed seronegative women
Source: PLoS One. 2020 Sep 1;15(9):e0238347. doi: 10.1371/journal.pone.0238347 (PMC7462289; doi:10.1371/journal.pone.0238347)

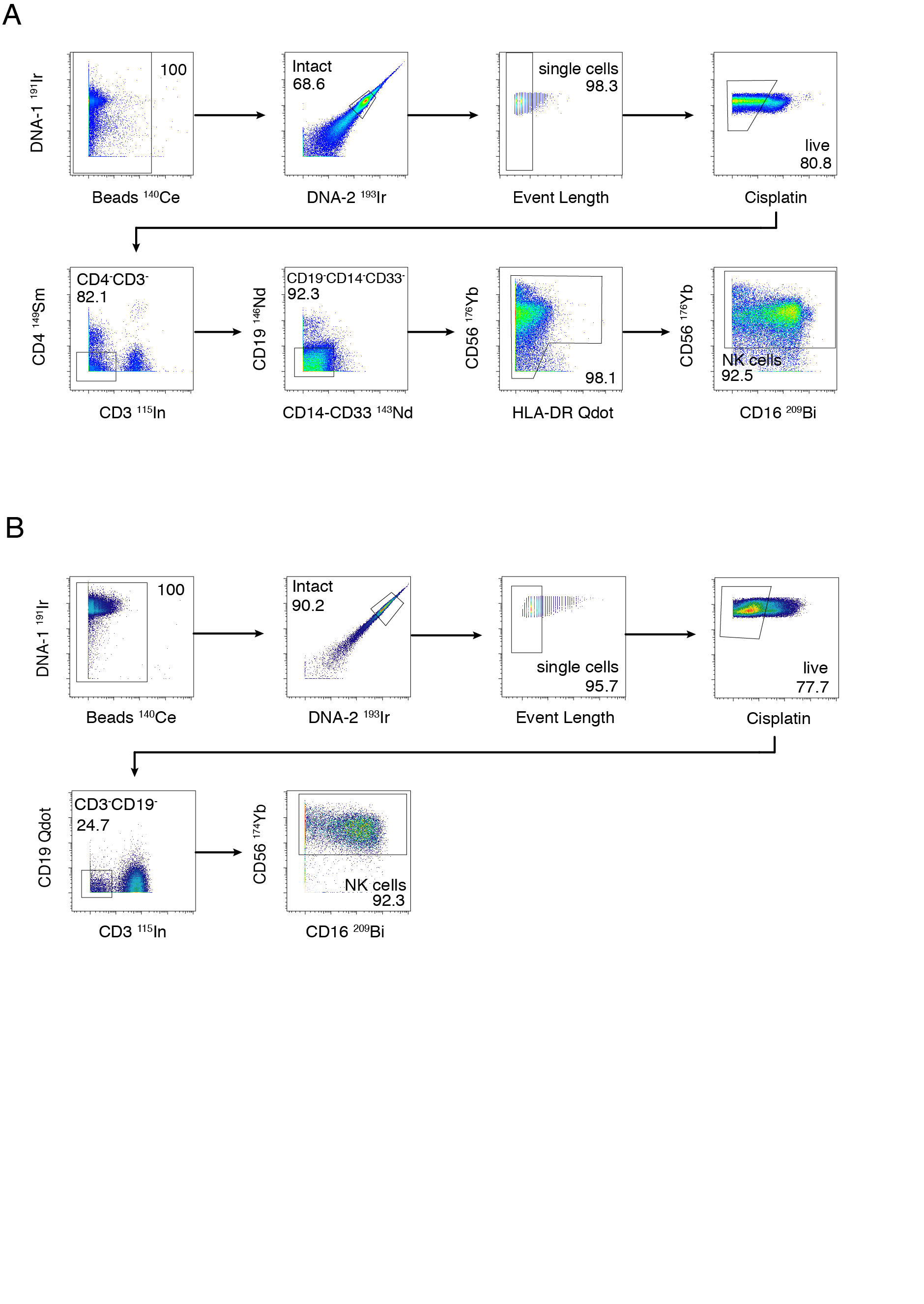

Supplement: S1 Fig — Intact, bead and event-length gates ensure successful gating to single cells. Cisplatin stain was performed as Live/Dead stain. (A) Serial negative gating to NK cells for CyTOF Panel 1. T cells and B cells were excluded using CD3, and CD19. Monocytes were excluded by negative gating on CD4 and CD14/CD33 and by further negative gating of CD56-/HLA-DRbright cells. CD56 and CD16 were used to identify NK cells. (B) Serial negative gating to NK cells for CyTOF Panel 2. T cells in the co-culture were excluded using CD3. CD56 and CD16 were used to identify NK cells. (TIF) [file pone.0238347.s001.tif]

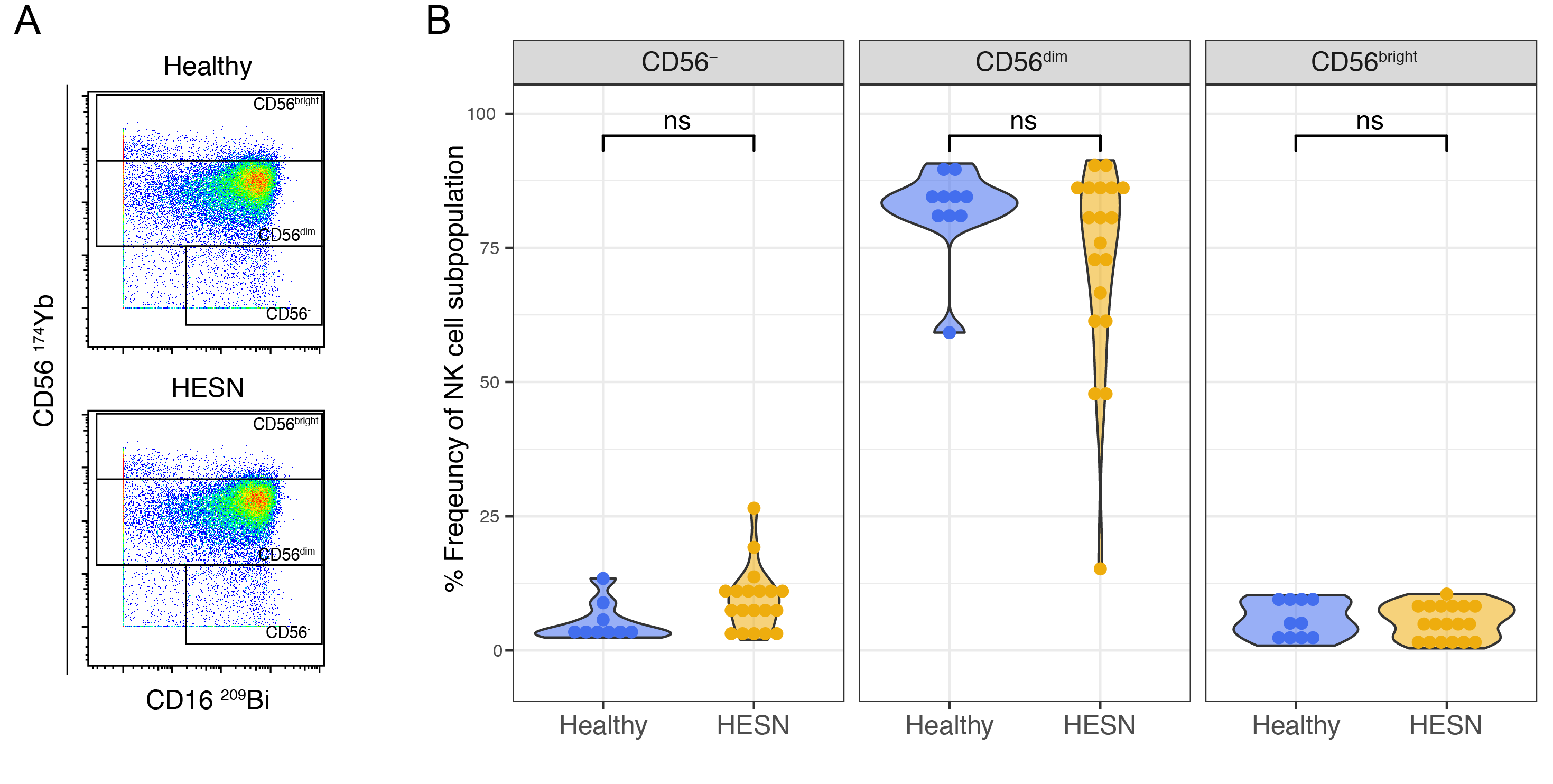

Supplement: S2 Fig — (A) An example of gating strategy for classical NK cell subpopulations (CD56-, CD56dim and CD56bright) in one healthy and one HESN woman. (B) Frequency of CD56-, CD56dim and CD56bright NK cells in healthy and HESN women. ns = non-significant. (TIF) [file pone.0238347.s002.tif]

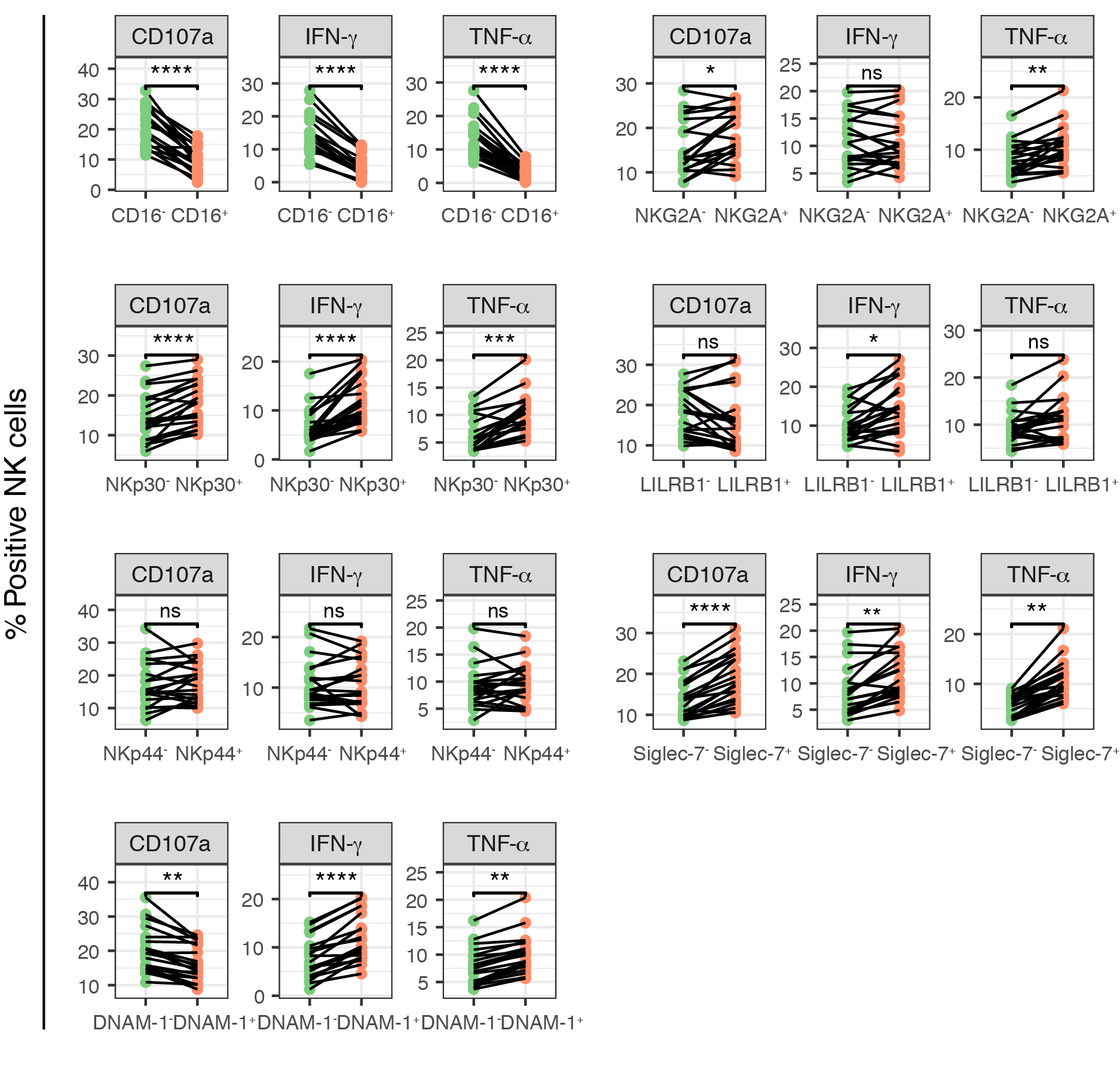

Supplement: S3 Fig — Summary data comparing the frequency of CD107a+, IFN-ɣ+ and TNF-ɑ+ NK cells of NKG2A+ and NKG2A-, NKp30+ and NKp30-, LILRB-1+ and LILRB1-, DNAM-1+ and DNAM-1-, SIglec-7+ and Siglec7-, and NKp44+ and NKp44- NK cells (n = 20). * = p ≤ 0.05 ** = p ≤ 0.01, *** = p ≤ 0.001, **** = p ≤ 0.0001, ns = not significant, by paired Wilcoxon signed-rank test, adjusted using the Benjamini-Hochberg method. (TIF) [file pone.0238347.s003.tif]

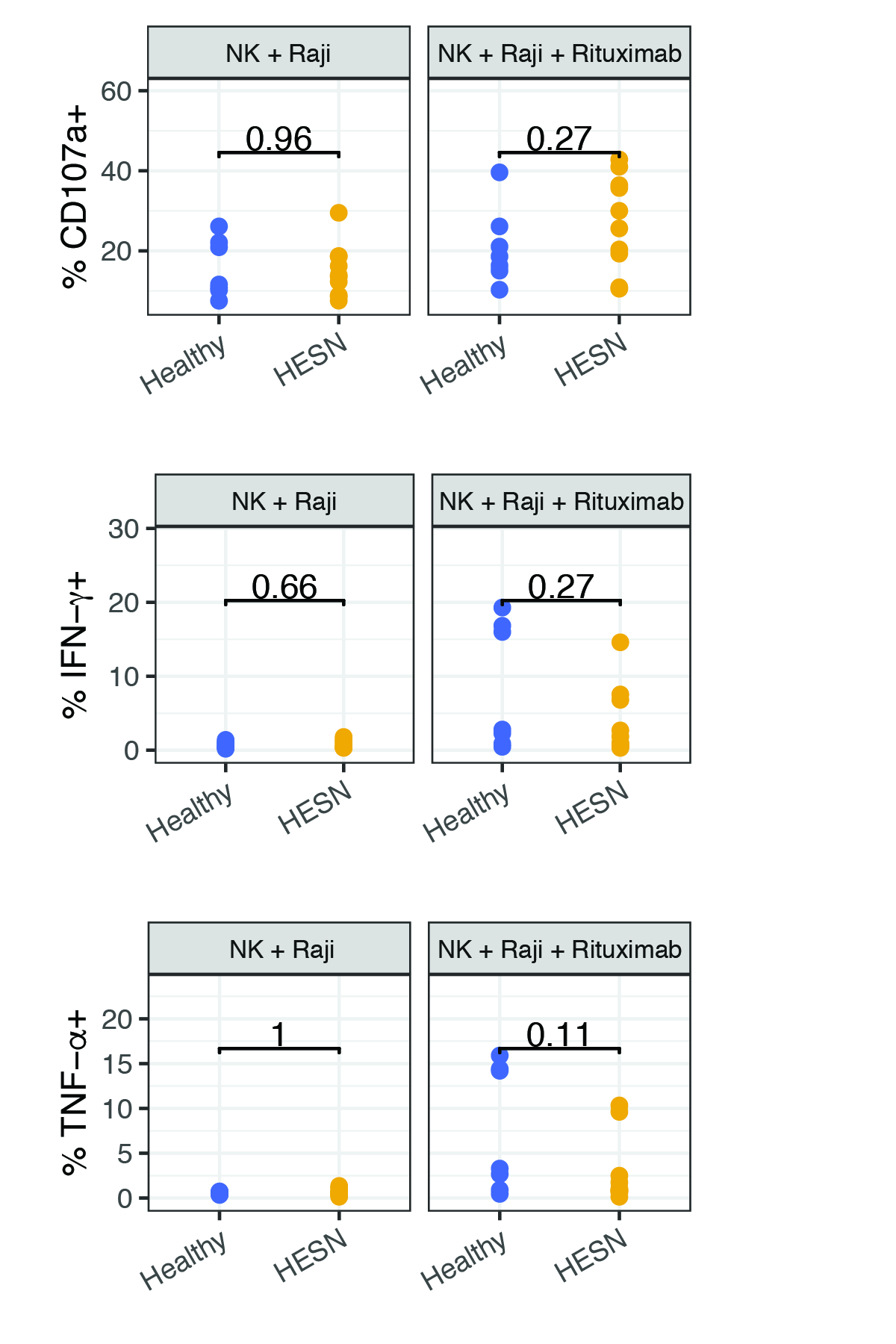

Supplement: S4 Fig — Frequency of cells positive for CD107a, IFN-γ, and TNF-ɑ in NK cells from healthy (n = 7) and HESN (n = 10) donors in an in vitro Rituximab-mediated ADCC assay with CD20+ Raji target cells, in the absence (left) or presence (right) of Rituximab (anti-CD20). Exact p-values by unpaired Wilcoxon signed-rank test are shown for each plot. (TIF) [file pone.0238347.s004.tif]

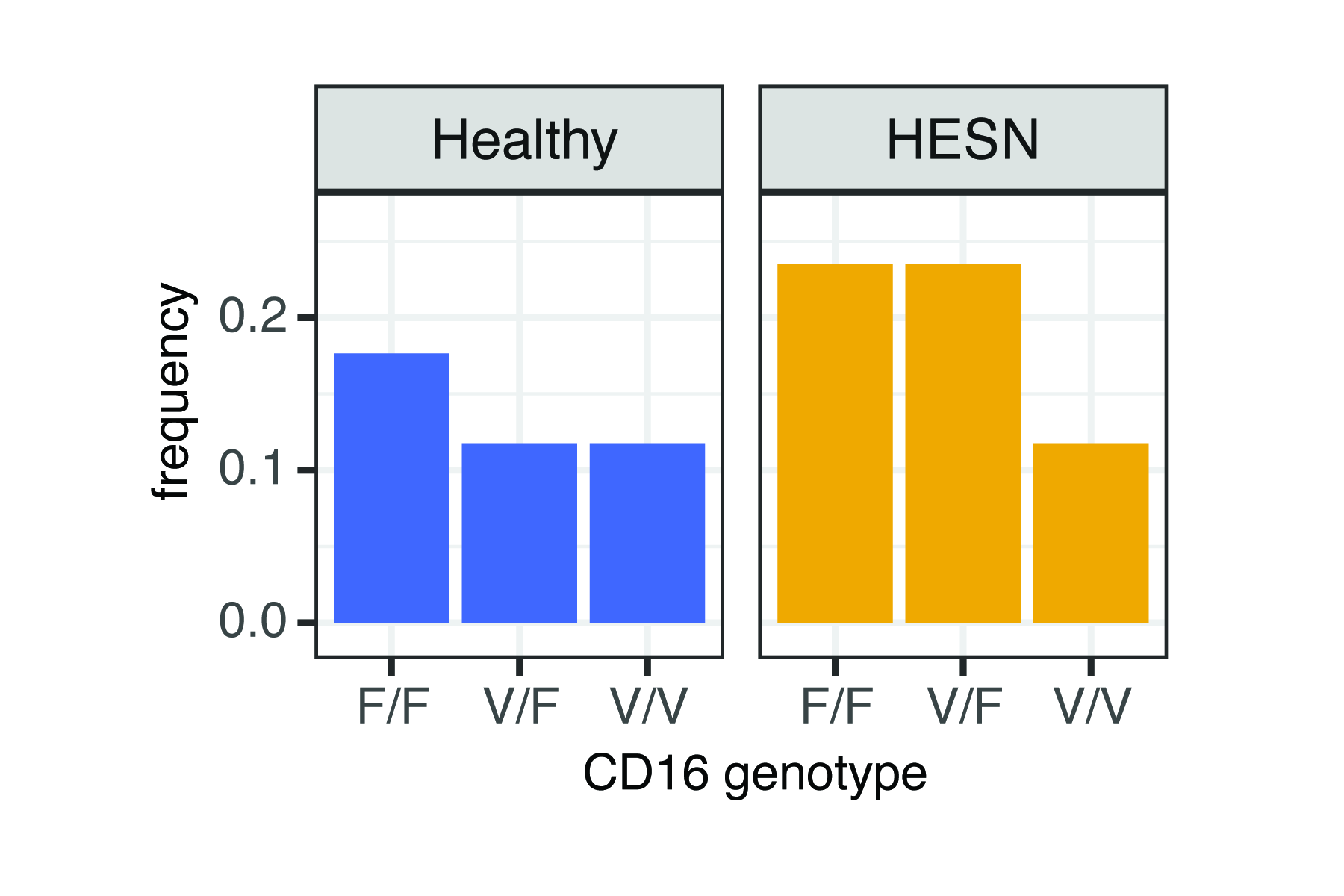

Supplement: S5 Fig — Frequency of each CD16 variant (F/F, V/F and V/V) in the healthy (n = 7) and HESN (n = 10) groups. Genotyping was performed by Sanger sequencing of the CD16 gene in the region containing the polymorphism. No significant difference in the frequencies between the two groups was found by Fisher’s exact test. (TIF) [file pone.0238347.s005.tif]
