# Supplementary material for: Natural killer cell phenotype is altered in HIV-exposed seronegative women
Source: PLoS One. 2020 Sep 1;15(9):e0238347. doi: 10.1371/journal.pone.0238347 (PMC7462289; doi:10.1371/journal.pone.0238347)
Supplement: S1 Table — (DOCX) [file pone.0238347.s006.docx]

**S1 Table. CyTOF Panel 1.**

| **Isotope** | **NK Marker** | **Source** | **Clone** | **Panel** |
| --- | --- | --- | --- | --- |
| ^89^Y | CD57 | Biolegend | HCD57 | Surface |
| ^112^Cd/Qdot | HLA-DR | Life Technologies | Tu36 | Surface |
| ^115^In | CD3 | Biolegend | UCHT | Surface |
| ^141^Pr | CD38 | Biolegend | HIT2 | Surface |
| ^142^Nd | CD69 | Biolegend | FN50 | Surface |
| ^143^Nd | CD33 | Biolegend | WM53 | Surface |
| ^143^Nd | CD14 | Biolegend | M5E2 | Surface |
| ^144^Nd | CD2 (LFA-2) | Biolegend | RPA-2.10 | Surface |
| ^145^Nd | LILRB1 (ILT-2/CD85j) | Biolegend | GHI/75 | Surface |
| ^146^Nd | CD19 | Biolegend | HIB19 | Surface |
| ^147^Sm | CD8 | Biolegend | SK1 | Surface |
| ^148^Nd | FcRɣ | Millipore | Polyclonal | ICS |
| ^149^Sm | CD4 | Biolegend | SK3 | Surface |
| ^150^Nd | Syk | Biolegend | 4D10.2 | ICS |
| ^151^Eu | CD62L | Biolegend | DREG-56 | Surface |
| ^152^Sm | Ki-67 | Biolegend | Ki-67 | ICS |
| ^153^Eu | KIR2DS4 | R&D Systems | 179315 | Surface |
| ^154^Sm | KIR2DS2 | Abcam | Polyclonal | Surface |
| ^155^Gd | NKp46 (CD335) | Biolegend | 9E2 | Surface |
| ^156^Gd | NKG2D | Biolegend | 1D11 | Surface |
| ^157^Gd | TIGIT | R&D Systems | 741182 | Surface |
| ^158^Gd | 2B4 (CD244) | Biolegend | C1.7 | Surface |
| ^159^Tb | DNAM-1 (CD226) | BD Biosciences | DX11 | Surface |
| ^160^Gd | FAS-L | Biolegend | NOK-1 | Surface |
| ^161^Dy | NKp30 (CD337) | Biolegend | P30-15 | Surface |
| ^162^Dy | Siglec-7 | Biolegend | S7.7 | Surface |
| ^163^Dy | NKG2C | R&D Systems | 134522 | Surface |
| ^164^Dy | NKp44 | Biolegend | P44-8 | Surface |
| ^165^Ho | CD96 (TACTILE) | Biolegend | NK92.39 | Surface |
| ^166^Er | KIR2DL1 | R&D Systems | 143211 | Surface |
| ^167^Er | CD94 | Biolegend | DX22 | Surface |
| ^168^Er | CXCR6 | Biolegend | K041E5 | Surface |
| ^169^Tm | PD1 (CD279) | Biolegend | EH12.2H7 | Surface |
| ^170^Er | KIR2DL5 | Miltenyi | UP-R1 | Surface |
| ^171^Yb | NKG2A | R&D Systems | 131411 | Surface |
| ^172^Tb | NTB-A | Biolegend | NT-7 | Surface |
| ^173^Yb | KIR3DL1 | BD Biosciences | DX-9 | Surface |
| ^174^Yb | CD56 | BD Biosciences | NCAM16.2 | Surface |
| ^175^Lu | KIR2DL3 | R&D Systems | 180701 | Surface |
| ^176^Yb | Perforin | Abcam | B-D48 | ICS |
| ^209^Bi | CD16 | Fluidigm | 3G8 | Surface |
