# Supplementary material for: Natural killer cell phenotype is altered in HIV-exposed seronegative women
Source: PLoS One. 2020 Sep 1;15(9):e0238347. doi: 10.1371/journal.pone.0238347 (PMC7462289; doi:10.1371/journal.pone.0238347)
Supplement: S2 Table — (DOCX) [file pone.0238347.s007.docx]

**S2 Table. CyTOF Panel 2.**

| **Isotope** | **NK Marker** | **Source** | **Clone** | **Panel** |
| --- | --- | --- | --- | --- |
| ^89^Y | CD57 | Biolegend | HCD57 | Surface |
| ^112^Cd/Qdot | CD19 | Invitrogen | SJ25-C1 | Surface |
| ^115^In | CD3 | Biolegend | UCHT1 | Surface |
| ^141^Pr | Granzyme B | Invitrogen | GB11 | ICS |
| ^142^Nd | MIP-1β | BD Biosciences | D21-1352 | ICS |
| ^143^Nd | NKG2C | R&D Systems | MAB1381 | Surface |
| ^144^Nd | CD161 (KLRB1) | BD Biosciences | DX12 | Surface |
| ^145^Nd | CD38 | Biolegend | HIT2 | Surface |
| ^146^Nd | CD8 | Biolegend | SK1 | Surface |
| ^147^Sm | CD107a (LAMP1) | Biolegend anti-APC | APC003 | ICS |
| ^148^Nd | LFA-1 (CD11a/CD18) | Biolegend | M24 | Surface |
| ^149^Sm | CD2 (LFA-2) | Biolegend | RPA-2.10 | Surface |
| ^150^Nd | HIV p24 core antigen | abcam | 39/5.4A | ICS |
| ^151^Eu | Siglec-7 | Biolegend | S7.7 | Surface |
| ^152^Sm | Perforin | abcam | B-D48 | ICS |
| ^153^Eu | KIR2DS4 (CD158i) | R&D Systems | 179315 | Surface |
| ^154^Sm | LILRB1 (ILT-2/CD85j) | R&D Systems | 292319 | Surface |
| ^155^Gd | NKp46 (CD335) | Biolegend | 9E2 | Surface |
| ^156^Gd | NKG2D | Biolegend | 1D11 | Surface |
| ^157^Gd | TIGIT | R&D Systems | 741182 | Surface |
| ^158^Gd | 2B4 (CD244) | Biolegend | C1.7 | Surface |
| ^159^Tb | DNAM-1 (CD226) | BD Biosciences | DX11 | Surface |
| ^160^Gd | IFN-γ | BD Biosciences | B27 | ICS |
| ^161^Dy | NKp30 (CD337) | Biolegend | P30.15 | Surface |
| ^162^Dy | TNF-ɑ | eBioscience | MAB11 | ICS |
| ^163^Dy | KIR3DL1 | BD Biosciences | DX9 | Surface |
| ^164^Dy | NKp44 | Biolegend | P44.8 | Surface |
| ^165^Ho | CD96 (TACTILE) | Biolegend | NK92.39 | Surface |
| ^166^Er | KIR2DL1 | R&D Systems | 143211 | Surface |
| ^168^Er | CD62L | Biolegend | DREG-56 | Surface |
| ^169^Tm | NKG2A | Fluidigm | Z199 | Surface |
| ^170^Er | KIR2DS2 | Abcam | Polyclonal | Surface |
| ^171^Yb | PD1 (CD279) | Biolegend | EH12.2H7 | Surface |
| ^172^Tb | NTB-A | Biolegend | NT-7 | Surface |
| ^174^Yb | CD56 | BD Pharmingen | NCAM16.2 | Surface |
| ^175^Lu | KIR2DL3 | R&D Systems | 180701 | Surface |
| ^176^Yb | CD69 | Biolegend | FN50 | Surface |
| ^209^Bi | CD16 | Fluidigm | 3G8 | Surface |
